# Effect of Hot-Pressing Temperature on the Properties of Eco-Friendly Fiberboard Panels Bonded with Hydrolysis Lignin and Phenol–Formaldehyde Resin

**DOI:** 10.3390/polym16081059

**Published:** 2024-04-11

**Authors:** Ivo Valchev, Viktor Savov, Ivaylo Yordanov, Stoyko Petrin, Petar Antov

**Affiliations:** 1 Faculty of Chemical Technologies, University of Chemical Technology and Metallurgy, 1757 Sofia, Bulgaria; ivoval@uctm.edu (I.V.); yordanov@uctm.edu (I.Y.); stpetrin@uctm.edu (S.P.); 2 Faculty of Forest Industry, University of Forestry, 1797 Sofia, Bulgaria; p.antov@ltu.bg

**Keywords:** fiberboard panels, bio-based adhesive, hydrolysis lignin, density, hot-pressing temperature

## Abstract

Lignin is the natural binder in wood and lignocellulosic plants and is regarded as the main natural and renewable source of phenolic compounds. Its incorporation in the composition of fiberboards will enhance both the environmental performance of the panels and the complex use of natural resources. In recent years, the increased valorization of hydrolysis lignin in value-added applications, including adhesives for bonding fiberboard panels, has gained significant research interest. Markedly, a major drawback is the retention of lignin in the pulp until the hot-pressing process. This problem could be overcome by using a small content of phenol–formaldehyde (PF) resin in the adhesive mixture as an auxiliary binder. The aim of this research work was to investigate and evaluate the effect of the hot-pressing temperature, varied from 150 °C to 200 °C, in a modified hot-press cycle on the main physical and mechanical properties of fiberboard panels bonded with unmodified technical hydrolysis lignin (THL) as the main binder and PF resin as an auxiliary one. It was found that panels with very good mechanical properties can be fabricated even at a hot-pressing temperature of 160 °C, while to provide the panels with satisfactory waterproof properties, it is necessary to have a hot-pressing temperature of at least 190 °C.

## 1. Introduction

The fiberboard industry is characterized by sustainable production levels, as the dry-process method is currently dominant [1]. In the dry process, the properties of the panels are mainly attributed to the adhesion bonds. Currently, the production of wood-based panels is dominated by synthetic, formaldehyde-based resins [2,3]. Notably, due to the environmental aspects of manufactured wood-based composites, the content of free formaldehyde in the panels is significantly limited, and from 2026, emission class E_0_ (formaldehyde content up to 4.0 mg/100 g) will be mandatory for all EU member states [4,5]. A viable solution to address this issue is the use of sustainable, formaldehyde-free, bio-based wood adhesives as partial or complete replacements for the commonly used thermosetting formaldehyde-based adhesives [6,7,8,9]. In recent years, lignin has attracted tremendous interest from both academia and industry as an abundant, renewable and environmentally friendly alternative to petroleum-based products for a number of end uses, including wood adhesives [10,11].

It should be noted that significant amounts of technical lignin, estimated to amount to approximately 100 million tons per year, are generated as a by-product of the pulp and paper industry, of which only about 2% is used for value-added applications, while the rest is primarily used as a fuel [12,13]. The types of industrial lignin, depending on the production method, are divided into Kraft lignin, organosolv lignin, hydrolysis lignin and lignosulfonates [8]. Kraft lignin is the most common type of technical lignin obtained on a commercial scale. Kraft lignin has been used for manufacturing water-soluble products, e.g., polyurethane, carbon fiber, flame retardants, etc. However, it is mainly burned in production facilities to obtain thermal energy and partially regenerate some chemical reagents. Organosolv lignin is obtained in very small amounts, and when using lignosulfonates as binders in the production of wood-based panels, significant deterioration of the waterproof properties of the materials is observed [14,15,16]. Technical hydrolysis lignin is obtained as a residual product during acid or enzymatic biomass hydrolysis to sugars to produce bioethanol or other valuable raw materials [17]. In Bulgaria, the plants located in Razlog and Dolna Mitropoliya process waste wood and agricultural lignocellulosic raw materials into sugars with an estimated yield of 38%. The production capacity is 12,000 t/y of yeast with 50% protein content and 600 t/y of furfural. The remaining technical hydrolysis lignin is deposited in the landfill between the towns of Bansko and Razlog. At the beginning of 2023, the available quantities of technical lignin in the landfill were about 200,000 tons. Waste hydrolysis lignin represents about 40% of the raw material in the hydrolysis process described above. This makes hydrolysis lignin a particularly promising candidate for wood adhesive applications.

**Figure 1:**
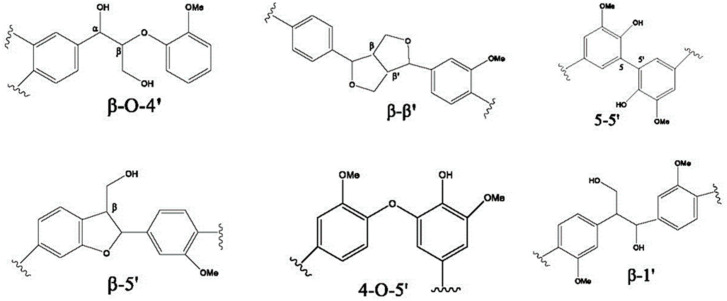
Different connections between lignin precursors [18].

In general, lignin is a natural and renewable biopolymer that plays a significant role as a binder in wood and lignocellulosic plants. The different types of linkages of the lignin precursors are presented in Figure 1 [18].

Figure 1 shows that surface-active groups are of primary importance for lignin in its capacity as a binder. Research in the field has proven that lignin is connected to cellulose mainly through ether bonds, acetal bonds and ester bonds. Ether and acetal bonds are formed between Cα of lignin and C6 of cellulose, while ester bonds are formed between Cγ of lignin and C6 of cellulose [19]. In general, lignin is not highly reactive, which, in some research in the field, is overcome through various modifications [20,21,22,23].

Another option is the modification of hot-pressing technology, applying initial low pressure, followed by increased pressure [24] and subsequent cooling [15,25]. One of the main problems with this approach is lignin retention in the wood fibers until it is plasticized and activated. This drawback can be successfully overcome by using an auxiliary binder [15,26]. Phenol formaldehyde (PF) resin is suitable for this purpose because of its use in the industrial manufacture of wood-based panels and because of the ability of lignin, under certain conditions, to bond with phenol [27].

In all these processes, the correct hot-pressing temperature is essential for optimizing lignin bonds, lignin–cellulose bonds, and overall fiberboard properties. It should be emphasized that increasing the hot-pressing temperature has a beneficial effect on the properties of fiberboard panels fabricated with standard formaldehyde-based resins [28,29]. However, the influence of hot-pressing temperature has not been investigated in detail when using a modified pressing cycle and an adhesive composition with hydrolysis lignin as the main binder and phenol–formaldehyde resin as the auxiliary, which determines the main aim and novelty of the present work.

## 2. Materials and Methods

### 2.1. Materials

Wood pulp fabricated by the “Asplund” (Stockholm, Sweden) thermomechanical method of refining was obtained from Welde-Bulgaria AD Troyan, Troyan, Bulgaria, and used to produce fiberboard panels. The pulp was composed of beech and Turkish oak in a ratio of 2:1. The supplied fibers were characterized by a bulk density of 29 kg·m^−3^ and a moisture content of 11.2%.

The phenol–formaldehyde (PF) resin used was produced by Prefere Resins Romania SRL (Rasnov, Romania) and also provided by Welde Bulgaria AD (Troyan, Bulgaria). The PF resin had the following main characteristics: solids content of 46%; viscosity of 358 MPa.s; and pH value of 6.8.

Batch technical hydrolysis lignin (THL), supplied by the depot in Razlog, Bulgaria, was used to carry out the experimental work. THL was a residual product from acid hydrolysis. Hydrolysis sugars and furfural were obtained in the production process, and were further subjected to chemical and biochemical processing. The process was carried out with diluted sulfuric acid at a concentration of 0.5–1%, and the duration of hydrolysis was 200–240 min at a maximum temperature of 190 °C. When taking the batch of THL from the landfill for the study, the surface layer of the lignin pile was removed to reduce the ash content. In previous studies conducted by the authors, the lignin from this batch was investigated regarding its potential applicability as biochar [30], with a view to using it for optimizing a modified hot-pressing cycle in the production of fiberboard panels with THL as a primary binder [15], and to assess the effect of the THL-to-PF resin ratio on the properties of eco-friendly panels [25]. Markedly, the effect of the hot-pressing temperature on the physical and mechanical properties of fiberboards with THL as a primary binder has not been investigated, which justifies the aim of the present research.

The chemical composition of THL was determined using previous methods of verifying the content of cellulose [31], lignin [32] and ash [33]. The C, N, S and H content was determined using a Euro EA 3000 Elemental Analyzer (EuroVector, Pavia, Italy). The results obtained for the chemical composition of the THL are presented in Table 1.

After fractionation, only hydrolysis lignin with a fraction below 100 μm was taken for the experiment.

In addition, the THL used was also characterized by Fourier transform infrared (FTIR) spectroscopy. Fourier transform infrared spectroscopy (FTIR) analysis was carried out using a Varian 600-IR (Palo Alto, CA, USA). For this purpose, 1 g of dry hydrolysis lignin was used. This study was conducted at the Laboratory of Thermomechanical and Thermophysical Analysis at the University of Chemical Technology and Metallurgy, Sofia, Bulgaria. The spectra were taken in the mid-infrared region of 400–4000 cm^−1^ with a resolution of 4 cm^−1^.

**Figure 2:**
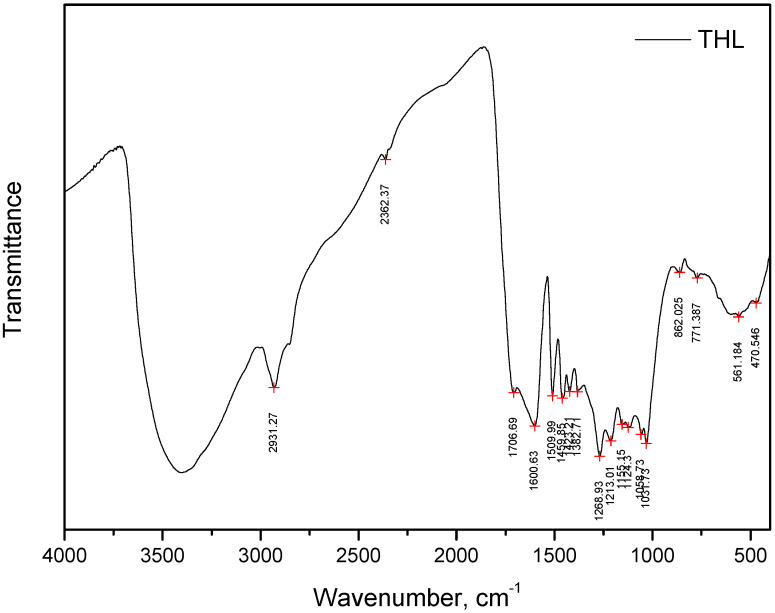
FTIR of technical hydrolysis lignin [30].

A graphical representation of the FTIR results is shown in Figure 2.

The FTIR data with the main functional groups in the technical hydrolysis lignin are presented in Table 2.

The binding functions of lignin are mainly the result of available O–H stretching (phenolic and aliphatic OH), C–H stretching (CH_3_ and CH_2_) and aromatic C–H in-plane deformation (S-ring). The comparative analysis of the absorption at spectra of 3391 cm^−1^ and 1382 cm^−1^ gives information about the relative ratio of phenolic to aliphatic OH [33]. This characteristic shows that in hydrolysis lignin, the most abundant hydroxyl group is phenolic OH. This observation is another reason for the better compatibility of lignin with PF resin compared to the other traditional formaldehyde-based synthetic resins used in the wood-based panel production industry, such as urea–formaldehyde or melamine–formaldehyde resin. 

**Figure 3:**
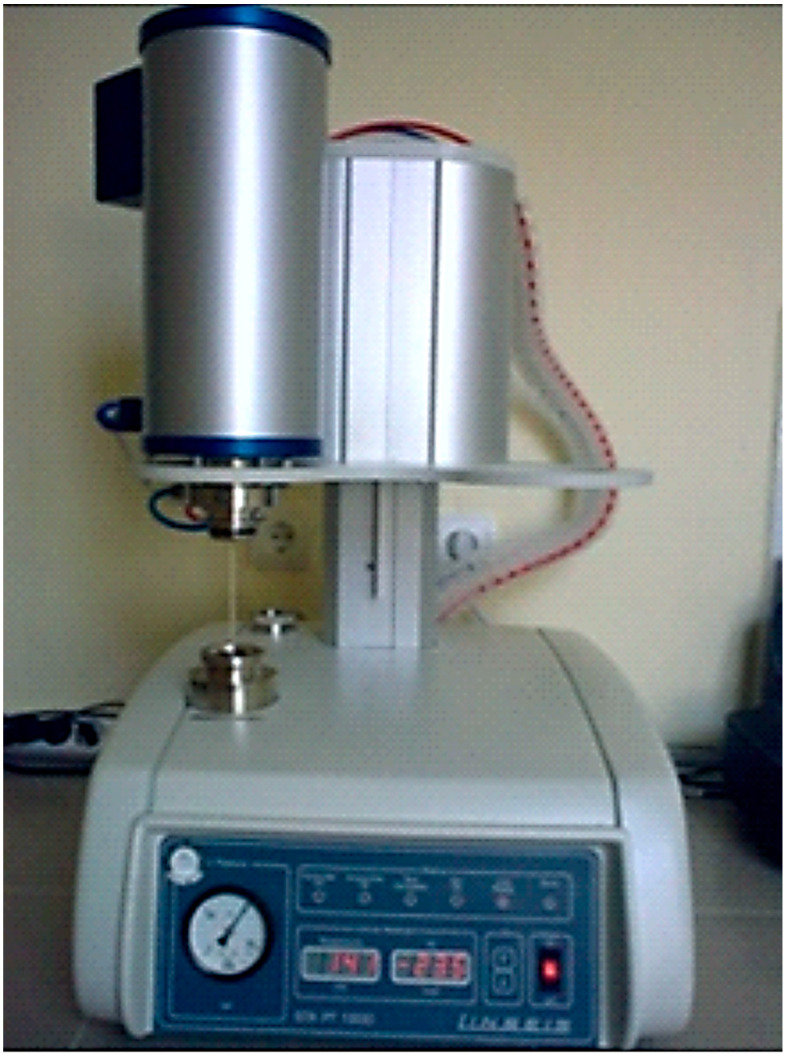
STAPT1600 TG-DTA/DSC apparatus.

Thermogravimetric analysis/differential thermogravimetry (TGA/DTG) was conducted using a STAPT1600 TG-DTA/DSC (STA Simultaneous Thermal Analysis) manufactured by LINSEIS Messgeräte GmbH, Selb, Germany, Figure 3.

**Figure 4:**
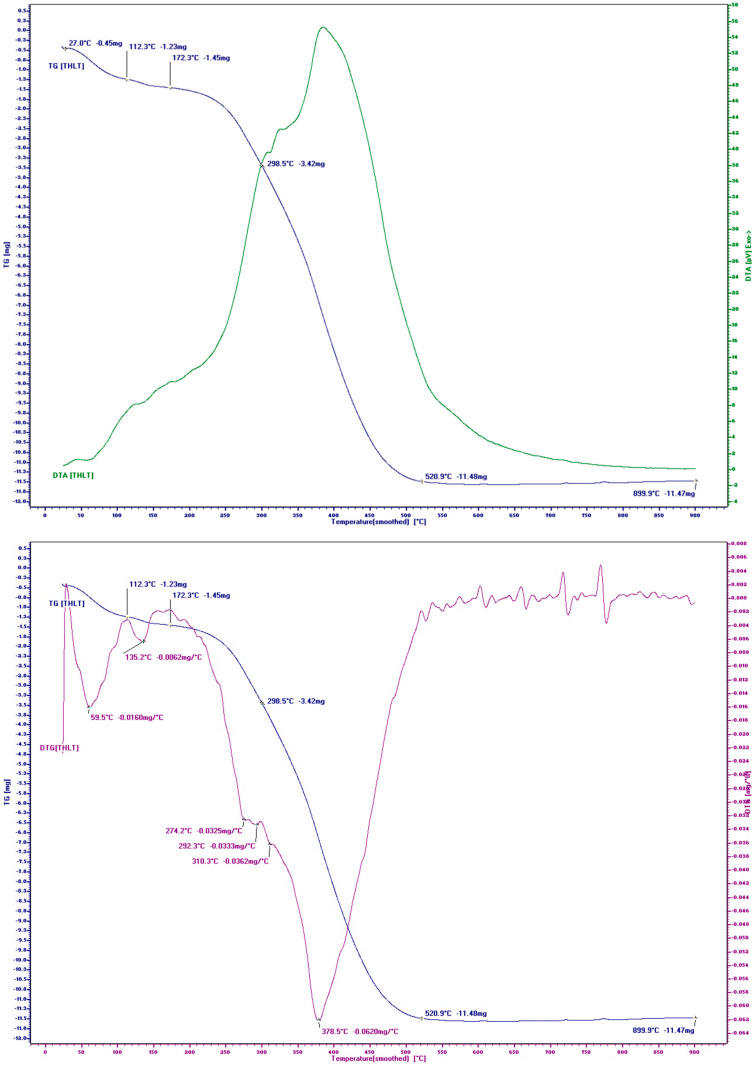
TG/DTG curves of technical hydrolysis lignin.

The parameters of this study were as follows: a temperature range of 20 ÷ 1000 °C; a heating rate of 10 °C/min; gas environment: static air environment; type of thermocouple: TypeS (Pt10%/Pt-Rh); types of crucibles: stabilized co-round crucibles. The thermogravimetric analysis/differential thermogravimetry (TGA/DTG) curves of THL are presented in Figure 4.

The TG/DTG curves of THL showed three stages of decomposition in the temperature range (30–1000 °C). The first stage (30–90 °C) was characterized by mass loss due to the evaporation of physically adsorbed moisture, and the peak temperature in this stage was 68.8 °C with a mass loss of about 25%. The second stage (90–200 °C) had a new peak at a temperature of 133.1 °C, which was mainly due to the evaporation of chemically bound water in the lignin, resulting in a mass loss of about 10%. The third stage (200–500 °C) showed that the actual phase transition of lignin began at a temperature of 200 °C, and at 378 °C, the lignin had already lost 80% of its mass. The organic elements of hydrolysis lignin burned completely at a temperature of 500 °C, and the inorganic residue was 8.45%. The glass-transition of technical hydrolysis occurred at 172.3 °C. 

### 2.2. Methods

The hot-pressing temperature was determined to vary from 150 °C to 200 °C, in steps of variation of 10 °C (Table 3). The panels had dimensions of 200 mm × 200 mm × 4 mm.

After determining the required amounts of materials, THL and PF resin were brought to a concentration of 30% of the solution and suspension, respectively. Then, they were mixed and immediately injected into the pulp. A fast-rotating laboratory blender (a prototype, University of Forestry, Sofia, Bulgaria) at 850 rpm with needle-shaped paddles was used. The adhesive system was injected through a nozzle with a diameter of 1.5 mm at a pressure of 0.4 MPa. The entire gluing process lasted 1 min. The hot-pressing process was carried out on a laboratory press “Servitec—Polystat 200 T” (Servitec Maschinenservice GmbH, Wustermark, Germany). The hot-pressing temperature varied from 150 °C to 200 °C. The press factor applied was 2 min·mm^−1^. A two-stage hot-pressing cycle with subsequent cooling was used. The first stage was at a pressure of 1.2 MPa and lasted 360 s. The second stage was at a pressure of 4.0 MPa and lasted 120 s. Cooling was carried out while maintaining high pressure (4.0 MPa) until a temperature below 100 °C was reached. In this case, the cooling time was 360 s. 

The laboratory-fabricated fiberboard panels were conditioned for two weeks at a room temperature of 20 ± 2 °C and a relative humidity of 65%.

The physical and mechanical characteristics of the fiberboard panels were determined in accordance with the applicable EN standards [35,36,37,38]. A universal testing machine, Zwick/Roell Z010 (ZwickRoell GmbH, Ulm, Germany), was used to determine the mechanical properties of the fiberboard panels.

## 3. Results and Discussion

### 3.1. Preliminary Results

**Figure 5:**
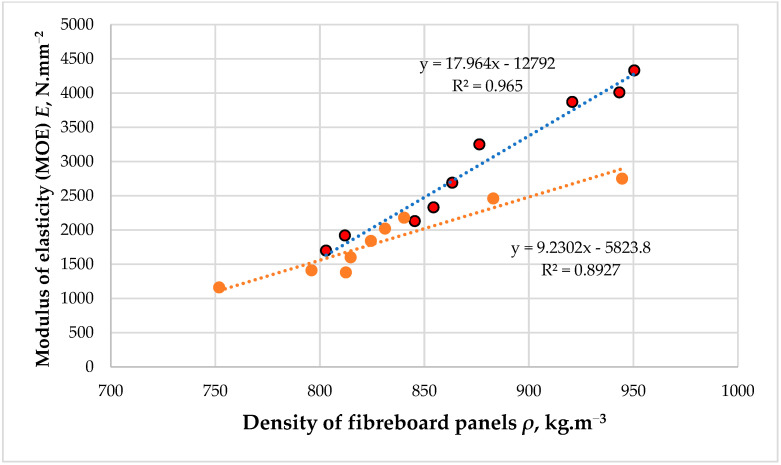
Modulus of elasticity (MOE) of fiberboard panels bonded with hydrolysis lignin as the main binder. Red dots represent the MOE values of the panels bonded with 7% THL and 3% PF resin. Orange dots represent the MOE values of the panels bonded only with 3% PF resin.

**Figure 6:**
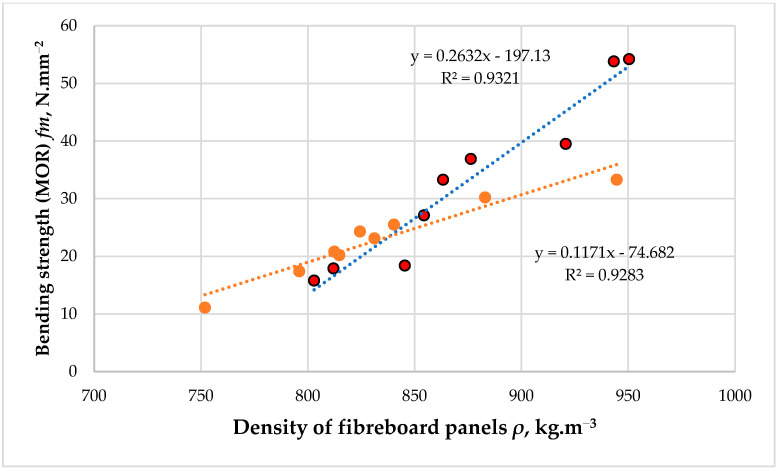
Bending strength (MOR) of fiberboard panels bonded with hydrolysis lignin as the main binder. Red dots represent the MOR values of the panels bonded with 7% THL and 3% PF resin. Orange dots represent the MOR values of the panels bonded only with 3% PF resin.

The preliminary studies carried out in laboratory conditions to fabricate eco-friendly fiberboard panels, bonded with hydrolysis lignin as the primary binder and a low PF resin content of 3% (based on the dry fibers), demonstrated a significant effect of panel density on the mechanical properties, i.e., the modulus of elasticity (MOE) and bending strength (MOR) of the laboratory-made fiberboards (Figure 5 and Figure 6).

The preliminary data on the effect of panel density on the main mechanical properties of the fiberboard panels clearly demonstrate that without further modification, the target density of the boards should be increased to activate the lignin to act as a binder.

Thus, at density values of about 800 kg·m^−3^, practically no action of the hydrolysis lignin as a binder was observed. The panels bonded with an adhesive system comprising 3% PF resin and 7% THL, and the panels bonded only with 3% PF resin exhibited similar MOE and MOR values. Under these conditions, the distances between the fibers are such that they do not form stable bonds with the hydrolysis lignin. When the target density of the panels was increased to 870–880 kg·m^−3^, bonds between the lignin and the fibers began to form. Thus, at these densities, the fiberboard panels bonded with hydrolysis lignin had nearly 1.3 times higher MOE and MOR values compared to the panels bonded with PF resin alone. This tendency was even more clearly expressed when the target density of the panels was set to 900 ÷ 950 kg·m^−3^. Thus, at a density of 950 kg·m^−3^, the fiberboards fabricated with THL exhibited 1.7 times higher MOE and MOR values.

The study of Mancera et al. [39] also confirmed the positive effect of lignin at increased fiberboard density, where fiberboard panels bonded with various types of unmodified lignin had 1.5–1.8 times higher mechanical and 1.4–2.0 times better waterproof properties than the panels without lignin. However, this was obtained at a relatively high fiberboard density of 1358 to 1380 kg·m^−3^. The positive effect of lignin addition at a panel density of 900 kg·m^−3^ was also confirmed by the study of Westin et al. [40], who reported that fiberboard panels fabricated with lignin had properties similar to those bonded with an 8% working solution of PF resin. Very good properties of fiberboard panels fabricated with different types of unmodified lignin were also reported by Tupciauskas et al. [41], but again, at significantly increased density values of about 1300 ± 50 kg·m^−3^. Velásquez et al. [42] also reported similar data when manufacturing fiberboard panels with a density that varied from 1200 to 1300 kg·m^−3^ using Kraft lignin as a binder.

Therefore, in the absence of lignin modification and without prior cross-linking with PF resin, the target density of the panels should be at least 900 ÷ 950 kg·m^−3^ in order to achieve satisfactory physical and mechanical properties of the composites produced. A subsequent increase in density above 1000 kg·m^−3^ is undesirable, given the need to use higher pressure during hot-pressing and the greater consumption of raw materials. Therefore, for the purposes of this research, namely establishing the effect of hot-pressing temperature on the properties of fiberboard panels fabricated with hydrolysis lignin as a primary binder, it was determined that the panels would have a target density of 950 kg·m^−3^.

### 3.2. Effect of Hot-Pressing Temperature

**Figure 7:**
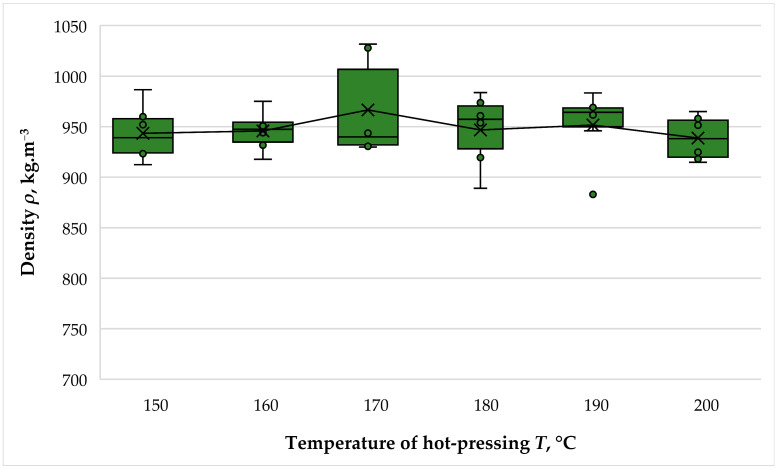
Density of fiberboard panels bonded with THL and PF resin.

The results obtained for the density of the fabricated panels are presented in Figure 7.

The density of the laboratory-produced panels varied from 939 kg·m^−3^ to 967 kg·m^−3^. That is, the difference in the maximum and minimum density values of the fiberboard panels was only 3.0%, or well below the statistical error of 5%. The density of the manufactured boards was very close to the target value of 950 kg·m^−3^. The conducted ANOVA, the results of which are shown in Table 4, also confirmed the non-significance of hot-pressing temperature on the fiberboard densities.

The absence of an influence of the hot-pressing temperature on the densities of the panels might be attributed to the selected method of hot-pressing, namely using metal bars to set the fiberboard thickness. The direct consequence of this is that the density of the panels is practically the same and will not reflect on the other physical and mechanical properties. Therefore, the variation in the other fiberboard properties could be due to the hot-pressing temperature.

Water absorption (WA) and thickness swelling (TS) are critical physical characteristics of wood-based panels, related to their dimensional stability, which provide important information on composite behavior in humid environments. 

**Figure 8:**
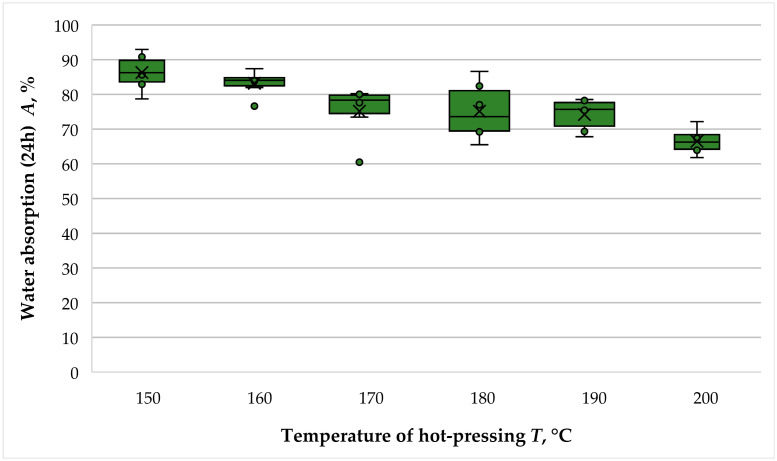
Water absorption (24 h) of fiberboard panels bonded with THL and PF resin.

The variation in the water absorption (WA) of fiberboard panels bonded with THL as the main binder as a function of hot-pressing temperature is presented in Figure 8.

As the hot-pressing temperature increased from 150 °C to 200 °C, the WA of the panels decreased from 86.31% to 66.51%, representing a total 1.3-fold decrease. However, in practice, two significant declines (improvements) in this property were observed. The first considerable decrease in WA occurred when the hot-pressing temperature was increased from 160 °C to 170 °C, resulting in 1.11 times better WA values. The second significant improvement in WA was determined when the hot-pressing temperature was increased from 190 °C to 200 °C, resulting in a 1.12-fold reduction in WA values.

The determined WA values at hot-pressing temperatures of 150 °C and 160 °C were almost the same. The conducted *t*-test also confirmed this observation, as the *p*-value was 0.260. The panels fabricated at temperatures of 170 °C, 180 °C and 190 °C had similar WA values, which was again confirmed by the conducted *t*-tests. The corresponding *p*-values were 1.000 and 0.812, respectively.

The determined effect of hot-pressing temperature on the WA values of the fiberboard panels was also consistent with the study of Wang et al. [43], where binderless fiberboard panels were fabricated. The cited research found a significantly smaller improvement in WA of about 1.08-fold as the hot-pressing temperature increased from 160 °C to 190 °C, and a 1.08-fold improvement again upon increasing the temperature of hot-pressing to 200 °C. Satisfactory WA values of the panels fabricated with different types of technical lignins at pressing temperatures in the order of 200 °C were also confirmed by the studies of Mancera et al. [39], Tupciauskas et al. [41] and Westin et al. [40]. Very good WA values of fiberboard panels at an elevated hot-pressing temperature of 230 °C were also reported by Theng et al. [44]. In the cited study, panels made with Kraft lignin resulted in 1.5 to 2.0 times lower WA values than commercial fiberboards. The need to increase the temperature of hot-pressing to the order of 200 °C to improve the WA of fiberboard panels bonded with hydrolysis lignin can be explained by the improved plasticization of lignin at these temperatures [45].

**Figure 9:**
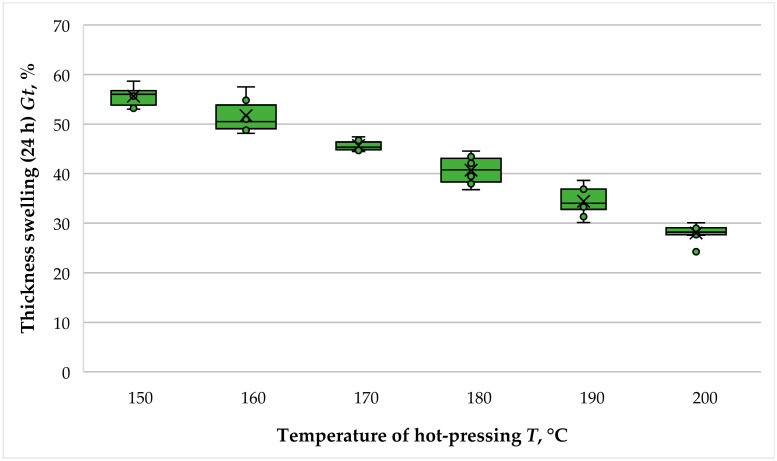
Thickness swelling (24 h) of fiberboard panels bonded with THL and PF resin.

The results obtained for the TS (24 h) of the fiberboard panels bonded with THL and PF resin are presented in Figure 9. 

The increase in the hot-pressing temperature from 150 °C to 200 °C resulted in significantly decreased TS values ranging from 56.64% to 28.01%, i.e., an 1.99-fold improvement.

The slightest improvement in TS values was determined when the pressing temperature was increased from 150 °C to 160 °C. In this case, the drop in TS was 1.08-fold. However, the improvement was statistically significant, which was confirmed by the conducted *t*-test (*p*-value of 0.055).

Subsequently, as the hot-pressing temperature increased from 160 °C to 190 °C, the TS was observed to improve (decrease) 1.13-fold as the temperature increased from 160 °C to 170 °C, 1.12-fold as the temperature increased from 170 °C to 180 °C and 1.18-fold when the temperature increased from 180 °C to 190 °C, respectively. The most significant improvement in TS values of 1.23 times was observed when the hot-pressing temperature was increased from 180 °C to 190 °C. Despite the substantial improvement in the property, due to the bio-based nature of the main binder used (technical lignin), only the panels fabricated at temperatures of 190 °C and 200 °C met the standard requirement for fiberboard panels with general purpose and use in dry conditions—a TS of, at most, 35%—and only the fiberboard panels fabricated at a hot-pressing temperature of 200 °C fulfilled the standard requirement for use in humid conditions, i.e., 30% [46].

The positive effect of increased hot-pressing temperature on the dimensional stability of fiberboard panels fabricated without the involvement of formaldehyde-based binders was also reported by Wang et al. [43]. In the cited study, increasing the hot-pressing temperature from 160 °C to 200 °C resulted in a 1.18-fold decrease (improvement) in TS values. Satisfactory TS values were also reported by Mancera et al. [39], Tupciauskas et al. [41] and Westin et al. [40]. Thus, the study of Westin et al., where the hot-pressing temperature was 190 °C, reported almost two times lower TS values of panels bonded with Kraft lignin compared to those made with PF resin. Tupciauskas et al. also reported satisfactory TS values of about 4% in fiberboard panels fabricated with lignin at ahot-pressing temperature of 235 °C. In the study by Mancera et al., using a hot-pressing temperature of 205 °C, panels bonded with THL exhibited about 1.5 times lower TS values than the control panels. In the study by Theng et al. [44], using a hot-pressing temperature of 230 °C, the fiberboards bonded with Kraft lignin had about two times better TS values than the control counterparts. 

Similar to the WA of the panels, the improvement in the TS of fiberboard panels made with technical lignin at increased hot-pressing temperatures can be explained by the plasticization of the lignin under these conditions (increased temperature and pressure).

**Figure 10:**
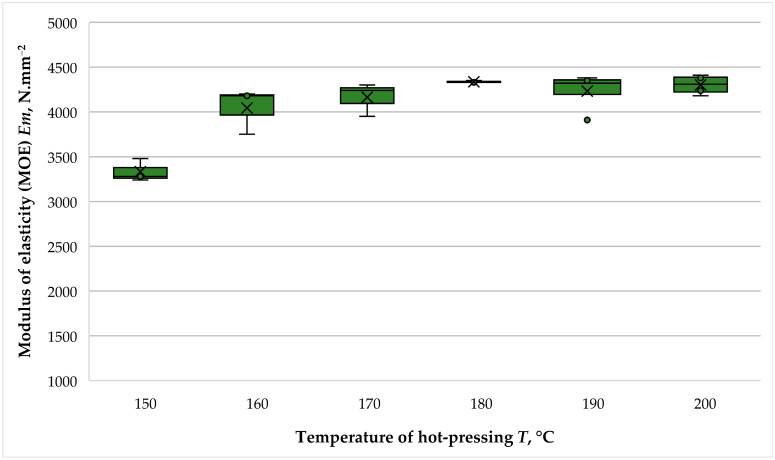
Modulus of elasticity (MOE) of fiberboard panels bonded with THL and PF resin.

The effect of hot-pressing temperature on the modulus of elasticity (MOE) of fiberboard panels fabricated with THL as a primary binder is presented in Figure 10.

The fiberboards bonded with hydrolysis lignin as a primary binder were characterized by relatively high MOE values. The laboratory-fabricated fiberboard panels produced in this work exhibited MOE values varying from 3332 N·mm^−2^ to 4337 N·mm^−2^. These high MOE values can be explained by the significantly extended compression factor of 2 min·mm^−1^. The lowest MOE value was recorded at a hot-pressing temperature of 150 °C, and the highest modulus was obtained for the panels fabricated at a hot-pressing temperature of 200 °C, i.e., the difference between the MOE values of the panels fabricated at these two temperatures was 1.30. However, it should be emphasized that the only significant difference in MOE was when the hot-pressing temperature was increased from 150 °C to 160 °C, resulting in a 1.22-fold increase in the property. The conducted *t*-tests showed that there was no statistically significant difference in the MOE of the panels with a subsequent increase in the hot-pressing temperature. Thus, the *p*-value at temperatures of 160 °C and 170 °C was 0.549. At hot-pressing temperatures of 170 °C and 180 °C, the *p*-value was 0.250; at 180–190 °C, the *p*-value was 0.410; and at temperatures of 190–200 °C, the *p*-value was 0.600. An explanation for this can be sought in the relatively extended press factor and the achievement of significant stiffness of the face layers of the panels even at a temperature of 160 °C.

All fiberboard panels fulfilled the strictest standard requirements for MOE, i.e., for use in load-bearing structures in humid conditions—a MOE of at least 3000 N·mm^−2^ [46].

Similar MOE values were reported in the study carried out by Wang et al. [43], who fabricated binderless fiberboard panels. In this research, an increase in the hot-pressing temperature from 160 °C to 200 °C resulted in a 1.66-fold improvement in MOE values. 

Very good MOE values of fiberboard panels fabricated with lignin at elevated hot-pressing temperatures were also reported by Mancera et al. [39], Tupciauskas al. [41] and Westin et al. [39]. Thus, in the study by Mancera et al., the fiberboards panels made with hydrolysis lignin had 1.25 times higher MOE values than the control panels. Tupciauskas et al. reported MOE values between 5000 and 7000 N·mm^−2^ for fiberboards with densities of 1300 kg·m^−3^ fabricated with lignin at hot-pressing temperatures of 235 °C. The study by Theng et al. [44] reported 1.2 times higher MOE values of fiberboard panels bonded with 9% lignin compared to commercial panels.

**Figure 11:**
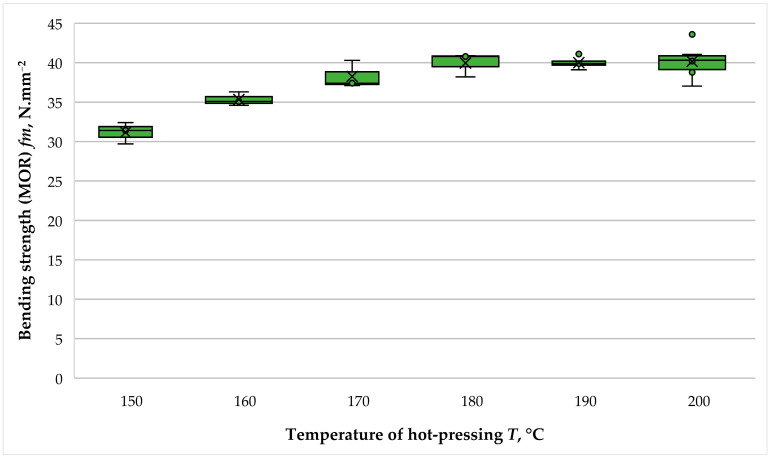
Bending strength (MOR) of fiberboard panels bonded with THL and PF resin.

The variation in the bending strength (MOR) of the panels with hydrolysis lignin as a primary binder depending on the hot-pressing temperature is given in Figure 11.

The MOR data significantly replicated the trends for the effect of hot-pressing temperature on the MOE of the panels. Overall, the fabricated fiberboard panels were characterized by a high MOR. Under the experimental conditions, this property changed from 31.17 N·mm^−2^ to 40.81 N·mm^−2^, or an overall 1.31-fold improvement was determined. Again, a significant difference in MOR values was observed between panels fabricated at a hot-pressing temperature of 150 °C and those manufactured at a hot-pressing temperature of 160 °C. The difference between the MOR values at these two temperatures was 1.14-fold. Unlike the MOE, in the MOR, a significant difference, i.e., a 1.08-fold improvement, was also observed when the hot-pressing temperature increased from 160 °C to 170 °C. An explanation here can be given by the fact that the MOE of the panels is only affected by the stiffness of the face layers. At the same time, the bending strength is partly influenced by the compressive resistance of the intermediate part of the panels (core layer). 

As a result of the conducted research, it was established that for fiberboard panels fabricated with THL as a primary binder and using a pressing factor of 2 min·mm^−1^, an increase in temperature above 170 °C during hot-pressing did not significantly affect MOR. The conducted *t*-tests confirmed this statement. Thus, the *p*-value for the MOR of panels fabricated at a hot-pressing temperature of 170 °C and 180 °C was 0.278. The corresponding values between the panels fabricated at hot-pressing temperatures of 180–190 °C and 190–200 °C were 0.975 and 0.490, respectively. 

Except for the panel fabricated at a hot-pressing temperature of 150 °C, all other fiberboards met the strictest standard requirements for the MOR, namely for load-bearing applications and use in humid conditions—a MOR of at least 35 N·mm^−2^ [46]. Even the panel fabricated at a hot-pressing temperature of 150 °C met the MOR requirement for load-bearing structures and use in dry conditions—a MOR of at least 29 N·mm^−2^ [46]. 

The MOR data obtained in the present study are consistent with the findings reported by Wang et al. [43] for the fabrication of binderless fiberboard panels. In this study, the MOR of the panels increased 1.46-fold as the hot-pressing temperature was increased from 160 °C to 200 °C. A real improvement in the MOR values was determined when the hot-pressing temperature was increased to 190 °C, followed by a negligible difference. The slight difference obtained in the present study might be attributed to the slightly extended press factor; hence, it can be concluded that in terms of MOR, it is not justified to increase the hot-pressing temperature above 180 °C. The determined trend of high MOR values of fiberboards fabricated at higher hot-pressing temperatures using lignin as a binder was also reported in previous research works [39,40,41,44].

**Figure 12:**
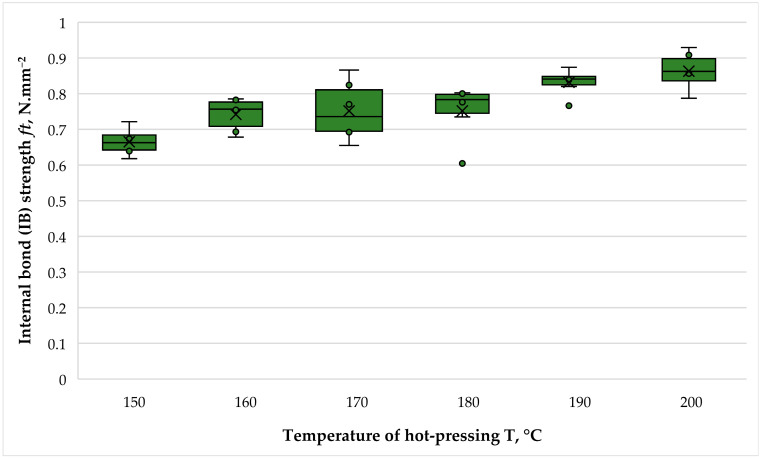
Internal bond (IB) strength of fiberboard panels bonded with THL and PF resin.

The variation in the internal bonds (IBs) of the fiberboard panels bonded with THL and PF resin is presented in Figure 12.

The IB refers to the bonding strength between fibers, which is of critical importance as it ensures that the fiberboards will not delaminate in post-processing. The internal bonding between wood fibers without the presence of synthetic adhesives is caused by the hydrogen bonds between the fibers, crosslinking between lignin and polysaccharides, and the condensation reaction of lignin [47,48,49,50]. Markedly, wood fibers with lignin-rich surfaces positively affect the mechanical properties of the composites due to the entanglement of the lignin caused by the hot-pressing temperature and pressure applied, and the enhancement of the formation of covalent bonds [51,52].

The IB values of the laboratory-made fiberboard panels varied from 0.67 to 0.86 N·mm^−2^, and the overall improvement in this property as the hot-pressing temperature increased from 150 °C to 200 °C was 1.30-fold. Two significant improvements in IB strength were observed, namely when the hot-pressing temperature was increased from 150 °C to 160 °C (a 1.12-fold improvement) and when the hot-pressing temperature was increased from 180 °C at 190 °C, i.e., an 1.11-fold increase. The conducted *t*-tests also confirmed the non-significance of the other values. The corresponding *p*-values were as follows: 160–170 °C—0.812, 170–180 °C—0.999, 190–200 °C—0.261. This observation leads to the conclusion that for a significant improvement in the core layer of the panels, the hot-pressing temperature should be increased to 190 °C. 

Except for the panels fabricated at a hot-pressing temperature of 150 °C, all other fiberboards fulfilled the strictest standard requirements for IB strength, namely for load-bearing applications and use in humid conditions—an IB strength of at least 0.70 N·mm^−2^ [46]. Even the panels fabricated at a hot-pressing temperature of 150 °C met the requirements for this property for panels with a general purpose and those use in humid conditions—0.65 N·mm^−2^ [46].

The improvement in the mechanical properties of the fiberboard panels, i.e., MOE, MOR and IB strength, with increased hot-pressing temperature were consistent with previous research works using lignin for bonding fiberboards [39,40,41,43,52]. The improvement in IB strength with increasing hot-pressing temperature could be attributed to the plasticization of lignin in the core layer of the panels, where failure occurs [45].

## 4. Conclusions

The present study confirmed that eco-friendly fiberboard panels with satisfactory water-related properties, that meet the standard requirements, and with excellent mechanical properties can be manufactured by using unmodified technical hydrolysis lignin and a low content of phenol–formaldehyde resin. As a result of the conducted preliminary experiments, a significant role of panel density in the activation of hydrolysis lignin as a binding substance was established. It was found that to utilize the adhesive abilities of lignin, the density of fiberboard panels should be about of 900 kg·m^−3^.

The main novelty of this study is that we conducted research on the effect of hot-pressing temperature on the properties of fiberboard panels bonded with hydrolysis lignin and a small amount of phenol–formaldehyde resin. Regarding this effect, with a variation range of hot-pressing temperature of 150–200 °C and a press factor of 2 min·mm^−1^, it was found that the optimal values of the factor were quite different regarding the waterproof and mechanical properties of the panels. Thus, to fabricate panels with very high mechanical properties, it is sufficient to use a hot-pressing temperature of 160 °C. Concerning the waterproof characteristics, especially for the thickness swelling of the panels, the hot-pressing temperature should be at least 190 °C, and in order to produce panels suitable for use in humid conditions, the hot-pressing temperature should be 200 °C.

Overall, this study confirmed one of the main disadvantages of bio-based binders—in this case, hydrolysis lignin—namely the difficulty of fabricating materials with good waterproof properties. Given the very good mechanical properties of the panels fabricated at not very high hot-pressing temperatures, the promising possibility of obtaining a structural eco-friendly material with reduced heat costs during production has been outlined. 

## Figures and Tables

**Table 1:** Chemical composition of technical hydrolysis lignin [15].

Lignin, %	Cellulose, %	Ash, %	C, %	S, %	H, %	N, %
72.6	25.5	2.8	55.54	0.74	7.10	0.26

**Table 2:** Functional groups in THL used in this work.

Band (cm^−1^)	Assignments [34]	THL
3412–3460	O–H stretching (phenolic and aliphatic OH)	3391
2960–2925	C–H stretching (CH_3_ and CH_2_)	2931
2850–2840	C–H stretching (OCH_3_)	2839
1705–1720	C=O stretching (unconjugated carbonyl)	1707
~1600	C=C stretching (aromatic skeleton) with C=O stretching	1600
~1513	C=C stretching (aromatic skeleton)	1510
1460	C–H deformation (asymmetric in CH_3_ and CH_2_) combined with C–H in-plane deformation	1459
~1425	C=C stretching (aromatic skeleton) with C–H in-plane deformation (aromatic skeleton)	1423
1365–1370	H–O in-plane deformation (phenolic OH) and C–H in methyl groups	1382
1266–1270	G-ring breathing with C–O stretching	1268
1215–1220	C–C with C–O and C=O stretching	1213
1166	C=O (conjugated ester groups)	1155
1115	Aromatic C–H in-plane deformation (S-ring)	1124
1030–1035	Aromatic C–H in-plane deformation with C–O deformation (primary alcohol and ether) and C=O stretching (unconjugated)	1031
853–858	C–H out-of-plane bending (C_2_, C_5_, and C_6_ of G-ring)	862

**Table 3:** Experimental plan.

Panel №	Temperature of Hot-Pressing *T*, °C
1.	150
2.	160
3.	170
4.	180
5.	190
6.	200

**Table 4:** ANOVA for the effect of hot-pressing temperature on the density of the panels.

Source of Variation	SS	df	MS	F	*p*-Value	F Crit
Hot-pressing temperature	2813.112	5	562.6224	0.50898	0.767169	2.533555
Error	33,161.73	30	1105.391			
Total	35,974.85	35				

## Data Availability

Data are contained within the article.
